# Research on the predictive effect of a combined model of ARIMA and neural networks on human brucellosis in Shanxi Province, China: a time series predictive analysis

**DOI:** 10.1186/s12879-021-05973-4

**Published:** 2021-03-19

**Authors:** Mengmeng Zhai, Wenhan Li, Ping Tie, Xuchun Wang, Tao Xie, Hao Ren, Zhuang Zhang, Weimei Song, Dichen Quan, Meichen Li, Limin Chen, Lixia Qiu

**Affiliations:** 1 Department of Health Statistics, School of Public Health, Shanxi Medical University, Taiyuan City, Shanxi Province China; 2 Endemic Disease Prevention and Control Section, Shanxi Center for Disease Control and Prevention, Taiyuan City, Shanxi Province China; 3 Department of Mathematical Statistics, School of Statistics, Jiangxi University of Finance and Economics, Nanchang, Jiangxi Province China; 4 Shanxi Provincial Peoples Hospital, Taiyuan City, Shanxi Province China

**Keywords:** Human brucellosis, ARIMA-ERNN model, ARIMA-BPNN model, Predictive effect

## Abstract

**Background:**

Brucellosis is a major public health problem that seriously affects developing countries and could cause significant economic losses to the livestock industry and great harm to human health. Reasonable prediction of the incidence is of great significance in controlling brucellosis and taking preventive measures.

**Methods:**

Our human brucellosis incidence data were extracted from Shanxi Provincial Center for Disease Control and Prevention. We used seasonal-trend decomposition using Loess (STL) and monthplot to analyse the seasonal characteristics of human brucellosis in Shanxi Province from 2007 to 2017. The autoregressive integrated moving average (ARIMA) model, a combined model of ARIMA and the back propagation neural network (ARIMA-BPNN), and a combined model of ARIMA and the Elman recurrent neural network (ARIMA-ERNN) were established separately to make predictions and identify the best model. Additionally, the mean squared error (MAE), mean absolute error (MSE) and mean absolute percentage error (MAPE) were used to evaluate the performance of the model.

**Results:**

We observed that the time series of human brucellosis in Shanxi Province increased from 2007 to 2014 but decreased from 2015 to 2017. It had obvious seasonal characteristics, with the peak lasting from March to July every year. The best fitting and prediction effect was the ARIMA-ERNN model. Compared with those of the ARIMA model, the MAE, MSE and MAPE of the ARIMA-ERNN model decreased by 18.65, 31.48 and 64.35%, respectively, in fitting performance; in terms of prediction performance, the MAE, MSE and MAPE decreased by 60.19, 75.30 and 64.35%, respectively. Second, compared with those of ARIMA-BPNN, the MAE, MSE and MAPE of ARIMA-ERNN decreased by 9.60, 15.73 and 11.58%, respectively, in fitting performance; in terms of prediction performance, the MAE, MSE and MAPE decreased by 31.63, 45.79 and 29.59%, respectively.

**Conclusions:**

The time series of human brucellosis in Shanxi Province from 2007 to 2017 showed obvious seasonal characteristics. The fitting and prediction performances of the ARIMA-ERNN model were better than those of the ARIMA-BPNN and ARIMA models. This will provide some theoretical support for the prediction of infectious diseases and will be beneficial to public health decision making.

## Background

Brucellosis is an anthropozoonosis caused by Brucella bacteria [1] and is also known as “Malta fever” [2, 3]. The main clinical manifestations are fever, sweating, muscle and joint pain, fatigue and other flu-like symptoms [4]. Because the early manifestations of human brucellosis are similar to the symptoms of flu, thus leading to early misdiagnosis and a lack of attention from patients, human brucellosis often develops into chronic brucellosis with serious complications [4, 5]. These complications impact human labour capacity to varying degrees, limit the development of farming and animal husbandry, affect the economic and trade development of countries and even the world, and cause serious disease burden and considerable economic losses [6–8], which are the main public health problems that seriously impact developing countries. Currently, there are approximately 170 countries or regions in the world affected by human brucellosis, accounting for 1/6 to 1/5 of the global population. Additionally, there are approximately 500,000 emerging cases each year, which seriously threatens people’s lives [9]. To date, there are approximately 350 million people affected by human brucellosis, and the incidence has exceeded the highest level in China. Located on the Loess Plateau, Shanxi Province is a mixed region of farming and animal husbandry and a typical epidemic area in northern China. The reported incidence ranked first for four consecutive years from 2000 to 2003, and the reported cases from 2004 to 2007 ranked in the top five in the country [10]. Human brucellosis not only adversely affects human health but also hinders the development of animal husbandry. Therefore, reasonable prediction is important for the prevention and control of human brucellosis.

At present, the time series prediction model is the most common method for predicting the epidemic trend of an infectious disease and is mainly divided into two categories. One category is the traditional prediction model, represented by the grey prediction model [11], the Markov model [12], the exponential smoothing method [13] and the autoregressive integrated moving average (ARIMA) model [14]. They achieve modelling and prediction by extracting linear information. Among them, the ARIMA model is the most popular method for infectious disease prediction and is used as a benchmark to evaluate many new modelling methods [15]. The other category is a prediction model based on machine learning theory that the nonlinear mapping performance is strong [16], such as Back Propagation Neural Network (BPNN) [17], Multivariate Adaptive Regression Splines (MARS) [18], Random Forest (RF) [19], Multilayer Perceptron (MLP) networks [20], Support Vector Machines (SVM) [20], and Radial Basis Function (RBF) [21], and has been used to predict the incidence of infectious diseases. Nevertheless, a major limitation of models such as BPNN and SVR is that they are intrinsically static; that is, they do not account for the dynamic nature of infectious disease sequences [22]. These static models can learn information only about the current time; they do not take advantage of historical information. The Elman recurrent neural network (ERNN) model obtains previous information through the receiving layer and can combine current information with historical information [23]. This characteristic makes it one of the most powerful tools for the prediction of nonlinear time series [24]. Time series is considered to consist of both linear and nonlinear components [25, 26]. Neither a single linear model nor a nonlinear model can capture the different patterns in the time series [27]. Both types of prediction models have problems with incomplete information extraction, and the prediction accuracy needs to be further improved.

In 1969, Bates J M and Granger C W J elaborated on the combined forecasting method, and their research results attracted great attention from researchers in related fields [28]. The combined model used the unique advantages of different models to analyse the characteristics of time series and achieve accurate prediction. In 2011, Khashei M et al. applied a combined model of ARIMA and an artificial neural network to time series prediction. The results showed that the combined model had better prediction performance than the single model [29]. In 2019, Li S et al. established a combined model of ARIMA and BPNN (ARIMA-BPNN) using coal consumption data in India. The research showed that the combined model had significantly higher prediction accuracy than the single model [30]. Currently, a combined model based on ARIMA and ERNN (ARIMA-ERNN) is mainly applied to air pollution prediction [31], spot price forecasting [32], error compensation [33] and other fields. Nevertheless, there have been no reports in the use of the combined model to predict the epidemic trend of human brucellosis. In this study, the ARIMA-ERNN model was established based on the monthly incidence data of human brucellosis from 2007 to 2017 in Shanxi Province and compared with the ARIMA-BPNN and ARIMA models to evaluate the fitting and predictive effects of the three models. This study will provide certain theoretical support for the prevention and control of human brucellosis in Shanxi Province and offer some reference for the prediction of infectious diseases.

## Methods

### Data sources

In this study, the reported cases of human brucellosis from January 2007 to December 2017 were obtained from Shanxi Provincial Center for Disease Control and Prevention. All cases were diagnosed under the ‘2007 Diagnostic Criteria of Brucellosis (WS269-2007)’ [34, 35]. Relevant demographic data were obtained from ‘the Statistical Yearbook of Shanxi Province’. The human brucellosis cases from January 2007 to December 2017 were assembled as monthly counts. The monthly incidence data of human brucellosis from January 2007 to December 2016 were used to build the ARIMA model. The fitted data of the ARIMA model were used as the input of neural networks and were split into two sections: a training set and a verification set. The training set data from January 2007 to December 2015 were employed to construct the neural network, and the verification set data from January 2016 to December 2016 were used to verify the neural network. The monthly incidence data from January 2017 to December 2017 were used as the test set to test the prediction performance of the three models.

### Analysis of seasonal characteristics

STL [36] can be used to decompose time series with seasonal characteristics into long-term trends, seasonal trends, and random effects as follows:

1 $$ {X}_t={T}_t+{S}_t+{I}_t $$

where *X*_*t*_ is the actual value of human brucellosis at time *t* and *T*_*t*_, *S*_*t*_ and *I*_*t*_ are the long-term trends, seasonal trends and random effects, respectively. Since STL is only suitable for the decomposition of the addition model, logarithmic or Box-Cox transformation is required for the multiplication model, and then the monthplot is used to identify the high-occurrence season of human brucellosis.

### ARIMA model

ARIMA [37], a classic model in many time series analyses, is usually constructed as ARIMA (p, d, q) (P, D, Q) _s_ as follows [23]:

2 $$ {\Theta}_P\left({B}^s\right){\theta}_p(B){\left(1-{B}^s\right)}^D{\left(1-B\right)}^d{x}_t={\Phi}_Q\left({B}^s\right){\phi}_q(B){w}_t $$

where Θ_*P*_, *θ*_*p*_, Φ_*Q*_ and *ϕ*_*q*_ are polynomials of order P, order p, order Q and order q, respectively. D and d represent the order of trend differencing and seasonal differencing, which are determined when the original time series is stable. p, q, P, Q and s represent the order of the autoregressive, moving average, seasonal autoregressive, seasonal moving average and seasonal periodicity, respectively, which are determined by the autocorrelation function (ACF) plot and the partial autocorrelation function (PACF) plot of the adjusted series. In this study, the monthly incidence of human brucellosis from January 2007 to December 2016 was used to build the ARIMA model, and the process included the following steps. First, the original series was smoothed with a differential method, and the Augmented Dickey-fuller (ADF) test was used to check the stationarity of the adjusted sequence. The white noise test method, also known as the Ljung-Box test, was used to determine whether the adjusted sequence was caused by random effects. If the *p* value was less than the significance level, the adjusted sequence was considered to be stationary and was not a random sequence. Second, the plots of the ACF and PACF of the adjusted sequence were used to provide a rough guide for reasonable models. Then, a test statistic was constructed to determine whether the residuals of candidate models were random effects, and maximum likelihood estimation (MLE) was used to perform the parameter test of the candidate models. At the same time, the Akaike information criterion (AIC), the Schwarz Bayesian information criterion (SBC) and the coefficient of decision (R^2^) were used to select the optimal model. When the AIC and SBC values of the models are relatively close, the model with the largest R^2^ is selected [38]. Finally, the incidence data from January 2017 to December 2017 were used to test the prediction effect of the optimal model.

### Artificial neural networks (ANNs)

ANNs [39] are nonlinear adaptive systems consisting of a large number of neural units. They are mainly used to establish an appropriate model by adjusting the connection weight between neurons to meet the requirements to solve practical problems. According to the different information flow directions of the neural network operation process, they can be divided into two basic forms: feedforward (static) neural networks and feedback (dynamic) neural networks. Therefore, two representative models, BPNN [40] and ERNN [23], are respectively used in this paper to establish the combined model.

BPNN is a classic multilayer feedforward neural network based on the error backpropagation algorithm and consists of an input layer, a hidden layer and an output layer. The neurons in the three layers are fully connected in order, while the neurons in the same layer are not connected, and the multilayer design enables them to mine more information and perform nonlinear mapping well. The essence of BPNN learning is to minimize the MSE between predicted and actual values by adjusting the connection weight between the input layer, the hidden layer and the output layer. The learning process is divided into two parts: forward propagation of information and backward feedback of errors. The information from the input layer through the hidden layer reaches the output layer, and the predicted value is obtained. When the error between the predicted and actual values does not satisfy the requirements, the error back propagation adjusts the connection weights of each layer and iterates the process until the requirements are met. The mathematical formulas of BPNN used in this study are shown as follows.

3 $$ {S}_j(t)={f}_1\left(\sum \limits_{i=1}^n\sum \limits_{j=1}^h{V}_{ij}{X}_i(t)\right) $$

4 $$ {Y}_k={f}_2\left(\sum \limits_{j=1}^h\sum \limits_{k=1}^o{W}_{jk}{S}_j(t)\right) $$

where *n*, *h* and *o* are the neuron numbers of the input layer, the hidden layer and the output layer, respectively. *X*_*i*_(*t*) is the input of the input layer at time *t*. *S*_*j*_(*t*) and *Y*_*k*_(*t*) are the outputs of the hidden and output layers, respectively. *V*_*ij*_(*i* = 1, 2, ⋯, *n*; *j* = 1, 2, ⋯, *h*) and *W*_*jk*_(*k* = 1, 2, ⋯, *o*) represent the connection weights of the input layer-the hidden layer and the hidden layer-the output layer, respectively. *f*_1_, *f*_2_ are activation functions of BPNN. With repeated learning, the model prediction accuracy is maximized. To obtain the most effective model, it is often necessary to define the model during the training process. In this paper, the hidden layer of BPNN selects the tan-sigmoid function, the output layer selects the linear function, the training function is trainlm, and the performance index is MSE. The parameters of the network are set to 10,000 iterations, the learning rate is 0.01, and the error is 0.004. The number of hidden layer neurons is calculated using the following empirical formula, where *a* is a constant between 1 and 10:

5 $$ h=\sqrt{n+o}+a $$

ERNN is a classical nonlinear local recursive network. In contrast to the feedforward neural network, the receiving layer is added to the hidden layer to achieve dynamic memory capabilities. ERNN consists of four parts: an input layer, a hidden layer, a receiving layer and an output layer. The input layer transmits signals. The hidden layer receives the input from the input layer and the feedback input of the receiving layer, and its self-joining mode has a strong sensitivity to time series data. The receiving layer stores the output value of the previous hidden layer and passes it to the current hidden layer by a one-step delay operator to achieve the purpose of dynamic memory. The output layer receives the output of the hidden layer, mainly the role of linear weighting. The learning process of ERNN is a process of learning and training sample data, obtaining dynamic characteristics between input and output parameters, and ultimately obtaining stable network parameters. In this paper, the training function is traingdx, the number of hidden layer neurons is also calculated by the above empirical formula, and other parameters are the same as the BP neural network. The mathematical formulas of ERNN used in this study are shown as follows:

6 $$ {S}_j(t)={g}_1\left(\sum \limits_{r=1}^h\sum \limits_{j=1}^h{U}_{rj}{S}_r\left(t-1\right)+\sum \limits_{i=1}^n\sum \limits_{j=1}^h{V}_{ij}{X}_i(t)\right) $$

7 $$ {Y}_k(t)={g}_2\left(\sum \limits_{j=1}^h\sum \limits_{k=1}^o{W}_{jk}{S}_j(t)\right) $$

Similar to the BPNN, *n*, *h* and *o* are the neuron numbers of the input layer, the hidden layer and the output layer, respectively. *X*_*i*_(*t*) is the input of input layer at time *t*. *S*_*r*_(*t* − 1), *S*_*j*_(*t*) and *Y*_*k*_(*t*) are the outputs of the receiving, hidden and output layers, respectively. *U*_*rj*_(*r* = 1, 2, ⋯, *h*; *j* = 1, 2, ⋯, *h*), *V*_*ij*_(*i* = 1, 2, ⋯, *n*) and *W*_*jk*_(*k* = 1, 2, ⋯, *o*) represent the connection weights of the receiving layer-the hidden layer, the input layer-the output layer and the hidden layer-the output layer, respectively. *g*_1_, *g*_2_ are activation functions of ERNN.

### ARIMA-BPNN model and ARIMA-ERNN model

The ARIMA model is suitable for extracting the linear components of the original time series, but it loses nonlinear information in the residual [41]. The nonlinear mapping ability of ANNs can reduce the error of the ARIMA model, so artificial neural networks based on the optimal ARIMA model are constructed to improve the prediction accuracy of the model. The specific steps are as follows: First, the optimal ARIMA model was established based on the original series, the fitting value was obtained, and the error was calculated by the following formula:

8 $$ {e}_t={y}_t-\overset{\wedge }{L_t} $$

where *y*_*t*_ is the actual value of the original series, $\overset{\wedge }{L_t}$ is the fitting value of the optimal ARIMA, and *e*_*t*_ is the error, also known as the residual. Since a first-order difference and a seasonal difference were performed in building the optimal ARIMA model, the incidence data of the first 13 months were lost in this step. Second, the data from February 2008 to December 2015 were used to build the BPNN or ERNN, and the data from January 2016 to December 2016 were used to verify the neural network. The input values were the fitting values of the optimal ARIMA model and the corresponding time information, and the actual values were taken as output. In this study, to avoid unnecessary results or difficult training processes causing algorithm convergence problems, we used the mapminmax function to normalize the input and output data [23]. Third, BPNN and ERNN continuously learned and trained the network through the input data set and the output data set, and we selected MSE as the evaluation index of network performance. When the MSE is the smallest, the corresponding BPNN and ERNN have the best fitting effect. Finally, the predicted values of the optimal ARIMA model from January 2017 to December 2017 were used as the input values of the combined model to obtain the output predicted values, and the inverse normalization method was used to restore the output predicted value of the combined models into meaningful data.

### Indicators of model performance

Three performance indexes, MSE, MAE and MAPE, are used to assess the fitting and prediction effects of those models. The smaller the value, the better the model performance.

9 $$ MSE=\frac{1}{N}\sum \limits_{K=1}^n{\left({X}_k-\overset{\wedge }{X_k}\right)}^2 $$

10 $$ MAE=\frac{1}{N}\sum \limits_{k=1}^N\left|{X}_k-\overset{\wedge }{X_k}\right| $$

11 $$ MAPE=\frac{1}{N}\sum \limits_{k=1}^N\frac{\left|{X}_k-\overset{\wedge }{X_k}\right|}{X_k}\times 100\% $$

*X*_*k*_ is the actual value at time *k*. $\overset{\wedge }{X_k}$ is the predicted value of the model. *N* is the number of the incidence data.

### Data analysis

STL was performed with the stl function of R statistical software version 3.1.2, the ARIMA model was built on the appropriate module of SAS Software version 9.2, and the combined model was built on MATLAB2014a.

## Results

### Seasonal characteristics of human brucellosis

**Fig. 1:**
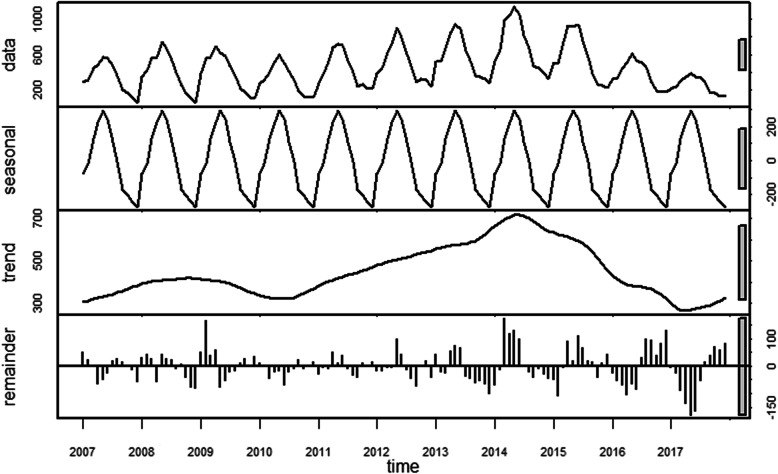
Seasonal decomposition based on STL of human brucellosis in Shanxi Province from 2007 to 2017

**Fig. 2:**
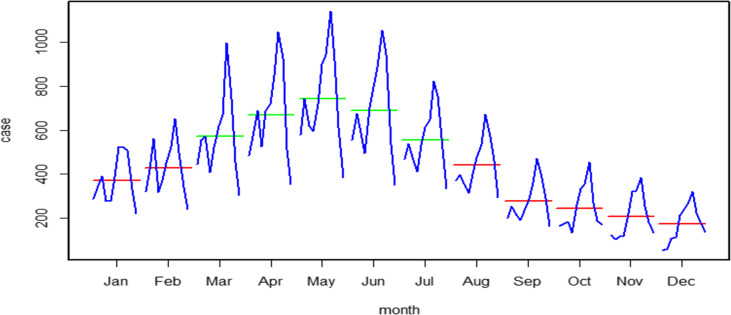
Monthplot of the cases of human brucellosis in Shanxi Province from 2007 to 2017

STL was used to study the time series of human brucellosis in Shanxi Province from 2007 to 2017, and the results are shown in Fig. 1. The grey bars of the figure represent the same magnitude and were used to compare the sizes of each part. The original data (data), seasonal trends (seasonal), long-term trends (trend) and random effects (remainder) are shown from top to bottom. Based on the seasonal part, human brucellosis in Shanxi Province showed obvious seasonality and periodicity, with a cycle of 1 year. The trend part revealed that the incidence increased from 2007 to 2014 and decreased from 2015 to 2017. However, the seasonal decomposition plot could not determine the peak season, which we solved by using the monthplot. We found that the long-term trend of reported cases was basically consistent in the same month of each year, and the data indicated that the months from March to July were high-risk months, of which the reported cases were the highest in May (Fig. 2).

### ARIMA model

**Fig. 3:**
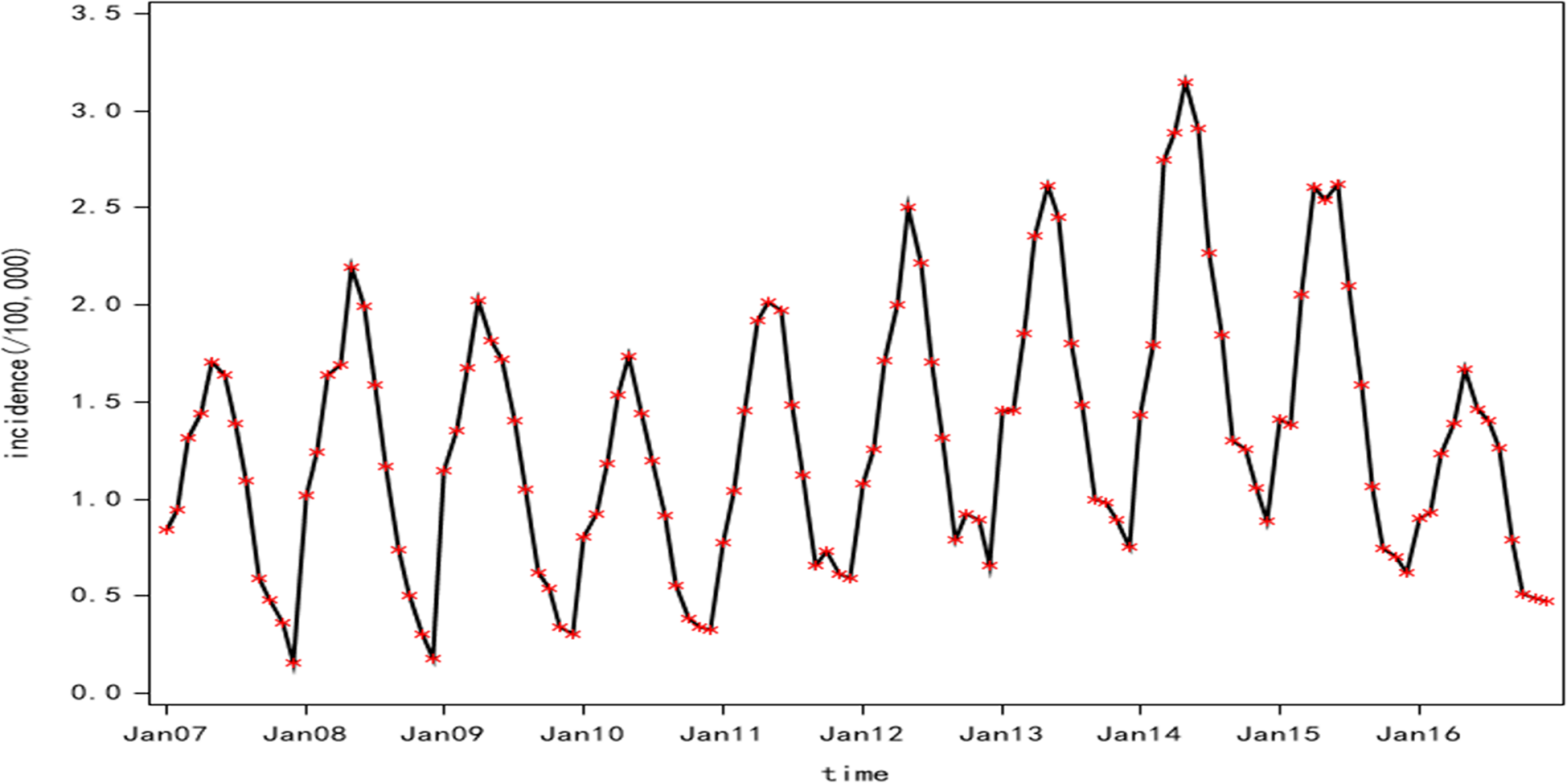
Time series plot for the incidence of human brucellosis in Shanxi Province from January 2007 to December 2016

**Fig. 4:**
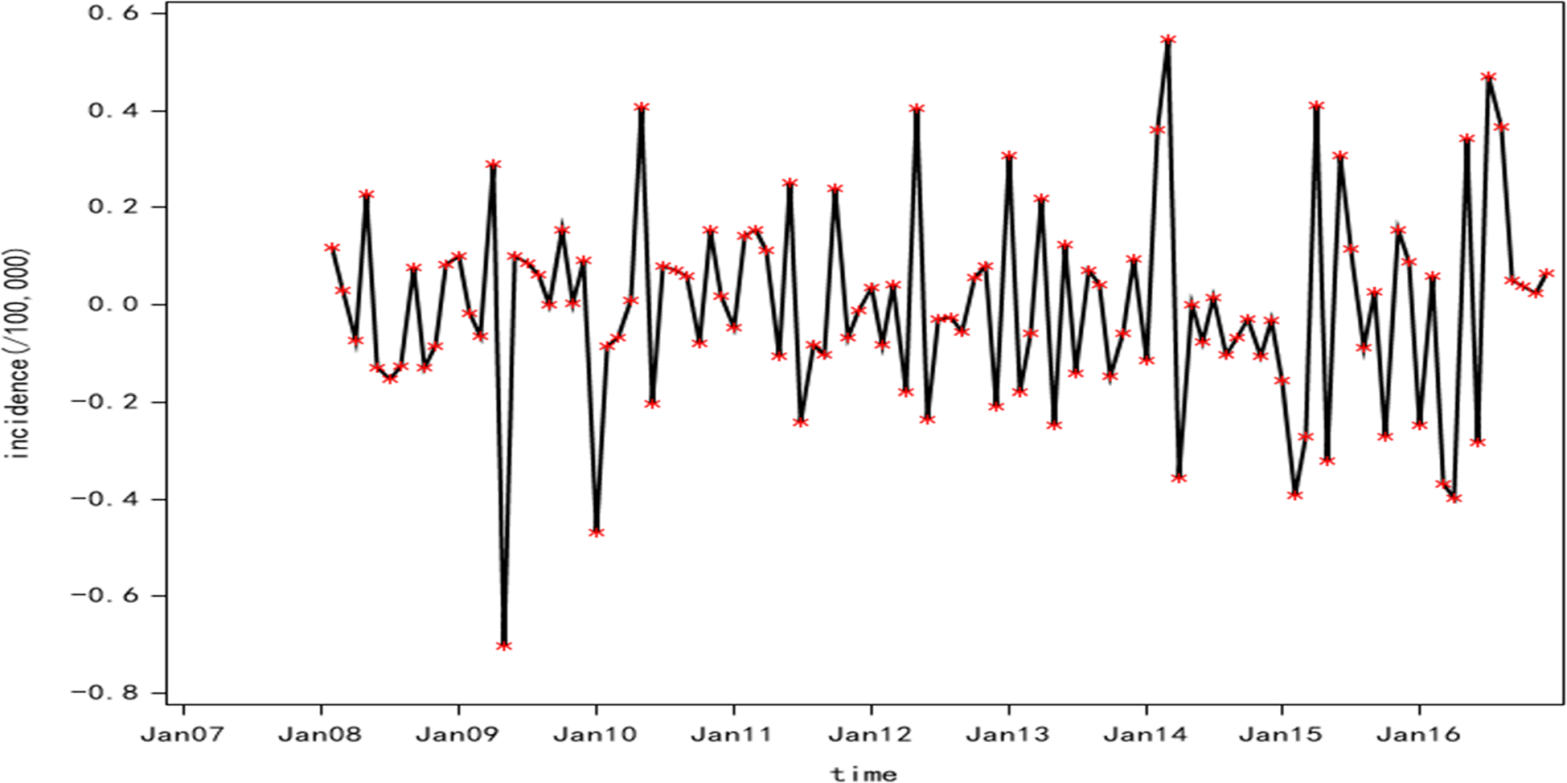
Plot of human brucellosis incidence after a first-order difference and a seasonal difference

**Fig. 5:**
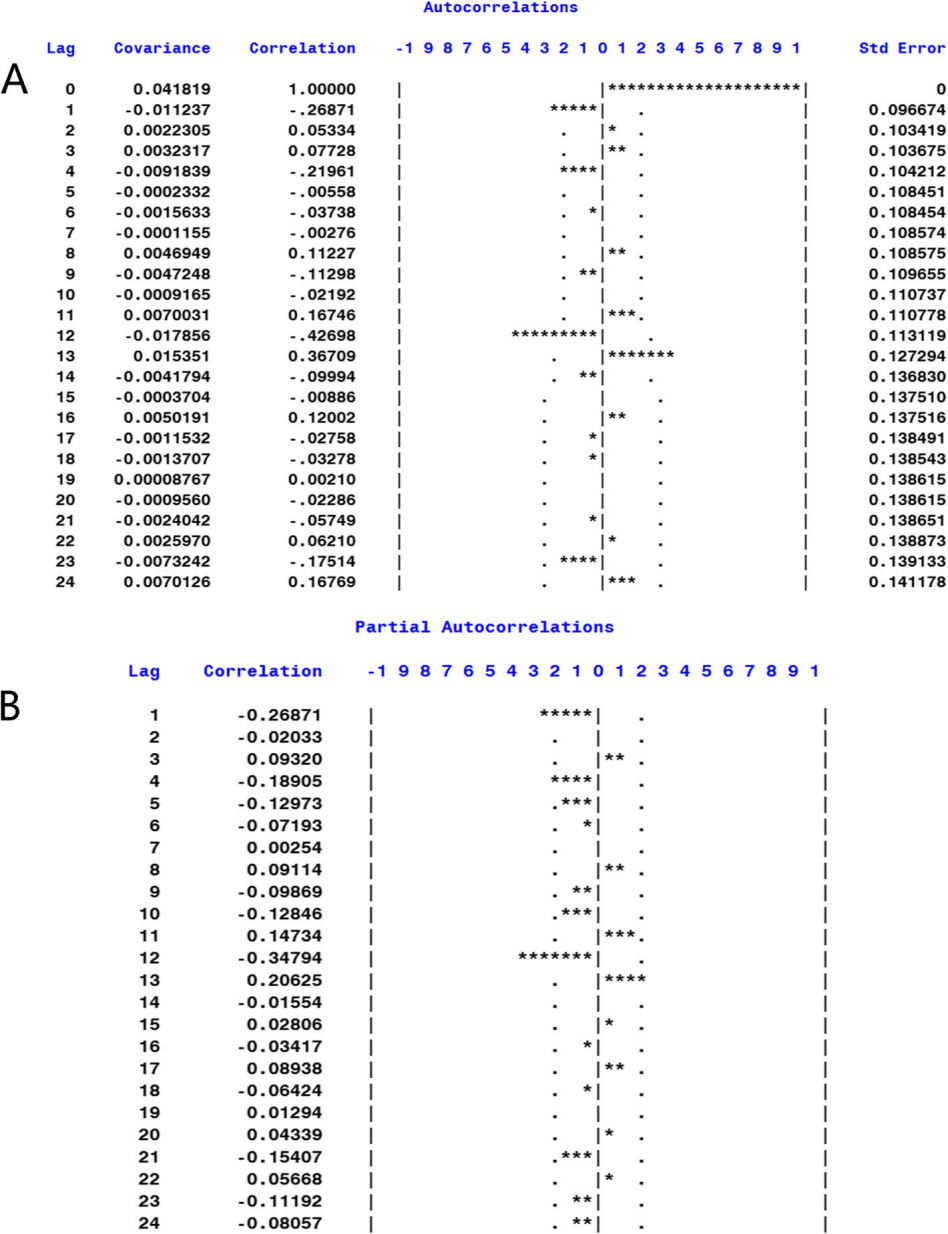
Autocorrelation and partial autocorrelation plots for the adjusted time series

The monthly incidence data of human brucellosis from January 2007 to December 2016 in Shanxi Province were used to develop the ARIMA model (Fig. 3). We also observed an upward trend from 2007 to 2014 and a significant decline from 2015 to 2016. The original series became stationary after the first-order difference and a seasonal difference, and the adjusted sequence was not a random effect (Fig. 4 and Table 1). The ARIMA model could be built at this time. Since the periodic change of the original series was 1 year, the parameters d, D and s for the ARIMA model were set to 1, 1 and 12, respectively. The possible values for P, Q, p, and q were determined according to the plot of ACF and PACF of the adjusted sequence (Fig. 5). The residual sequence of those fitting models was a random sequence (Table 2). Therefore, several alternative models could be initially identified by the residual test:

**Table 1:** ADF and Ljung-Box tests of the time series

Time series	ADF Test		Ljung-Box Test	
	T	*P*	χ^2^	*P*
Original series	−2.86	0.179	749.83	< 0.001
Adjusted series	−13.51	< 0.001	72.73	< 0.001

**Table 2:** Selection of the optimal model from among the four candidate models

Candidate models	Parameter estimate				Fitting index			Ljung-Box Test	
	AR1	SAR1	MA1	SMA1	AIC	SBC	R^2^	χ2	*P*
ARIMA (1,1,0) (1,1,0)_12_	−0.0914	− 0.3839*	–	–	− 363.82	− 358.47	0.929	18.42	0.6807
**ARIMA (0,1,1) (0,1,1)**_**12**_	**–**	**–**	−0.7900*	−0.9913*	**− 374.14**	**−368.79**	**0.938**	**21.60**	**0.4843**
ARIMA (1,1,1) (1,1,0)_12_	−0.2288	−0.3835*	0.1363	–	− 361.82	− 353.80	0.929	18.43	0.6217
ARIMA (1,1,1) (0,1,1)_12_	−0.3295	–	−0.2707	0.6712*	−374.03	− 366.00	0.937	24.04	0.2909

*P ≤ 0.05. The residuals of the four candidate models were tested using the Ljung-Box Test

ARIMA (1,1,0) (1,1,0)_12_, ARIMA (0,1,1) (0,1,1)_12_, ARIMA (1,1,1) (1,1,0)_12_, and ARIMA (1,1,1) (0,1,1)_12_.

The MLE was used to estimate the parameters of the candidate model. According to the results of the parameter estimates and fitting index, we found that the parameters of the ARIMA (0, 1, 1) (0, 1, 1)_12_ model were statistically significant and that the residual sequence of the model was a random sequence. In addition, the AIC and the SBC of this model were the smallest, and the R^2^ was the largest (Table 2). Therefore, the ARIMA (0, 1, 1) (0, 1, 1)_12_ model was the optimal model for prediction.

### ARIMA-BPNN model and ARIMA-ERNN model

According to the above formula, the hidden layer neurons of the BPNN and ERNN were between 3 and 14. We tried different neuron numbers in the hidden layer (Table 3) and found that when the numbers of hidden layer neuron in the BPNN and ERNN were 7 and 11, respectively, the performance of the two models was optimal; that is, the structure of BPNN was 2–7-1, and the structure of ERNN was 2–11-1. Finally, the predicted values of the ARIMA (0,1,1) (0,1,1)_12_ model from January 2017 to December 2017 were used as the inputs of BPNN with a structure of 2–7-1 and ERNN with a structure of 2–11-1, respectively, and the output values were the predicted values of the combined models.

**Table 3:** Training error of the ARIMA-BPNN and ARIMA-ERNN models

Neuron number of the ARIMA-BPNN model	MSE	Neuron number of the ARIMA-ERNN model	MSE
3	0.0133	3	0.0115
4	0.0109	4	0.0099
5	0.0147	5	0.0107
6	0.0144	6	0.0112
**7**	**0.0101**	7	0.0123
8	0.0131	8	0.0095
9	0.0123	9	0.0091
10	0.0135	10	0.0114
11	0.0120	**11**	**0.0088**
12	0.0112	12	0.0093
13	0.0133	13	0.0107
14	0.0140	14	0.0128

### Comparison of the three models

**Fig. 6:**
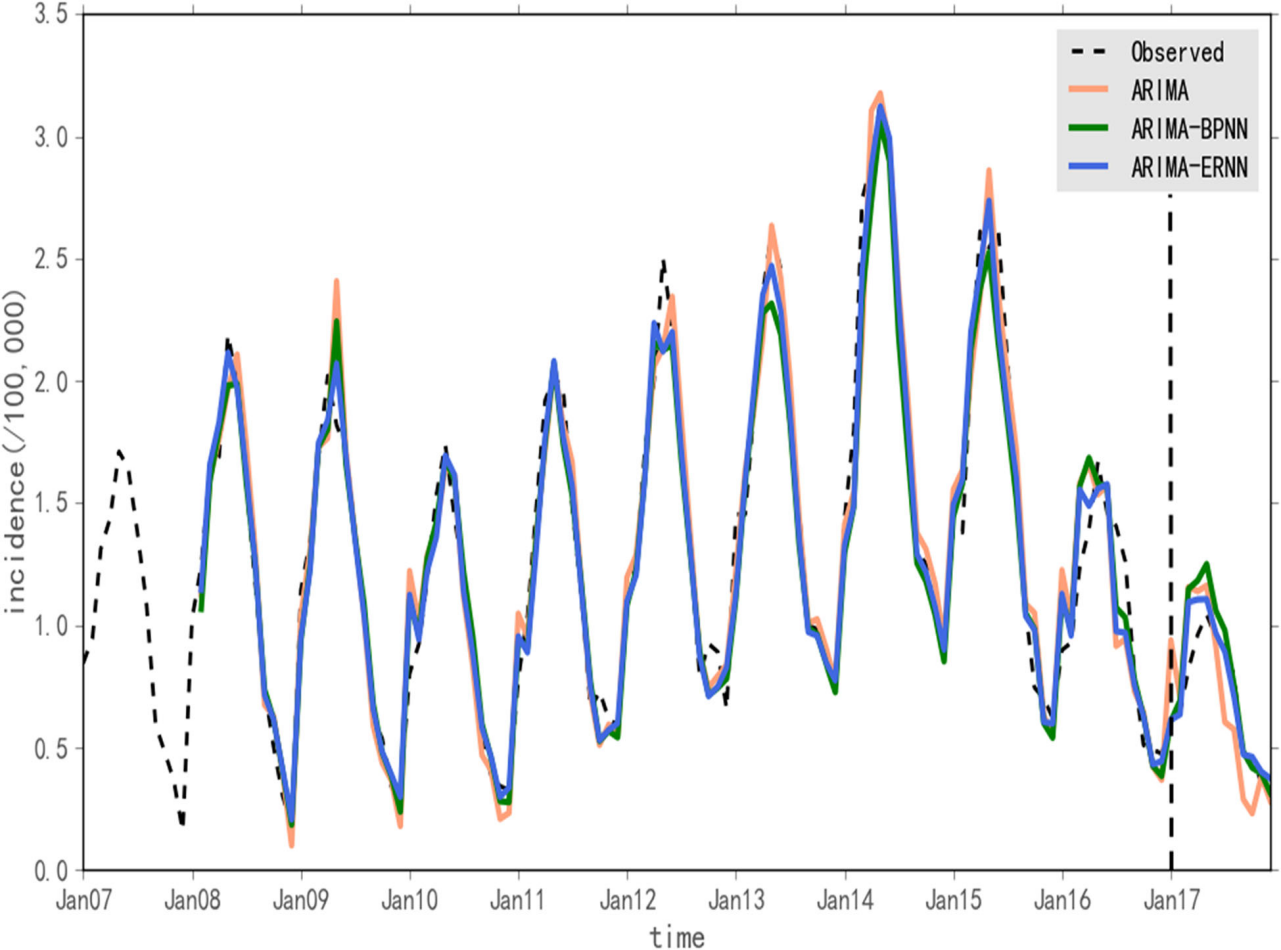
Predictive values obtained by using the ARIMA, ARIMA-BPNN and ARIMA-ERNN models and the incidence of human brucellosis in Shanxi Province. The figure is divided into two parts by a dashed line. The left side of the figure is the fitting part, and the right side is the prediction part

The optimal ARIMA model, the ARIMA-BPNN model and the ARIMA-ERNN model were used to predict the incidence of human brucellosis in Shanxi Province from January 2017 to December 2017. The predicted values of the three models and the incidence of human brucellosis are shown in Fig. 6. The fitting and prediction performances of the three models were compared by MSE, MAE and MAPE (Table 4). The combined model was better than the single ARIMA model, and the ARIMA-ERNN model was better than the ARIMA-BPNN model.

**Table 4:** Comparison of the three models in fitting and prediction performance

Model	Fitting performance			Prediction performance		
	MAE	MSE	MAPE (%)	MAE	MSE	MAPE (%)
ARIMA	0.1319	0.0305	12.01	0.1728	0.0417	26.23
ARIMA-BPNN	0.1187	0.0248	10.45	0.1006	0.0190	13.28
ARIMA-ERNN	**0.1073**	**0.0209**	**9.24**	**0.0688**	**0.0103**	**9.35**

Compared with those of the ARIMA model, the MAE, MSE and MAPE of the ARIMA-ERNN model decreased by 18.65, 31.48 and 64.35%, respectively, in fitting performance; in terms of prediction performances, the MAE, MSE and MAPE decreased by 60.19, 75.30 and 64.35%, respectively. Compared with those of the ARIMA model, the MAE, MSE and MAPE of the ARIMA-BPNN model decreased by 10.08, 16.68 and 12.53%, respectively, in fitting performance; in terms of prediction performance, the MAE, MSE and MAPE decreased by 41.78, 54.44 and 49.37%, respectively. Compared with those of the ARIMA-BPNN model, the MAE, MSE and MAPE of the ARIMA-ERNN model decreased by 9.60, 15.73 and 11.58%, respectively, in fitting performance; in terms of prediction performance, the MAE, MSE and MAPE decreased by 31.63, 45.79 and 29.59%, respectively.

## Discussion

Since 2000, with the rapid development of agriculture and the animal husbandry economy in Shanxi Province, human brucellosis has become one of the fastest-growing infectious diseases in Shanxi Province [42]. The incidence of human brucellosis in Shanxi Province showed an upward trend from 2007 to 2014 and peaked in 2014 (Fig. 3). This may be related to the implementation of the Central Transfer Payment Brucellosis Prevention and Control Project since 2006 and the pilot project of human brucellosis prevention and treatment from 2008 to 2010. In the early stage, the persistence of risk factors for human brucellosis and increased awareness of brucellosis among residents and medical institutions led to an increase in epidemiology reporting. The incidence decreased yearly from 2015 to 2017, which may be due to the successful implementation of the above two initiatives [43].

The analysis of seasonal characteristics (Fig. 1) shows significant seasonal characteristics, mainly in the period from March to July. The main reason may be related to farming operations. In the spring, herders have close contact with livestock because of shearing. Summer is the peak season of delivery of livestock such as cattle and sheep, which greatly increases the chances of contact with pathogenic factors during this process [44]. Moreover, most human brucellosis infections occur in spring and summer, which are attributed to meteorological and temperature factors. As the temperature and relative humidity decrease in autumn and winter, the survival rate of pathogenic bacteria decreases, thereby reducing the chance of infection in humans [45]. Therefore, prevention and control measures for brucellosis should consider seasonal fluctuations, and some targeted interventions should be performed at the peak of the epidemic. This suggests that we should pay special attention to protection when we are in contact with cattle, sheep and other livestock and implement active monitoring measures.

Accurate prediction of epidemic trends is of great significance for the prevention and control of human brucellosis [19]. The occurrence of brucellosis is subject to many factors, and it is difficult to collect data on influencing factors. However, the time series prediction model can overcome the shortcomings of conventional mathematical-statistical methods in the face of this situation, and all the complex external factors are attributed to the time factor to predict the future incidence. The ARIMA model is one of the most commonly used methods in infectious disease prediction and has been proven to have high accuracy [38]. It does not require additional variables and is more practical when the data for other influencing factors are not available. In this paper, we used the optimal model ARIMA (0, 1, 1) (0, 1, 1)_12_ as the basic model for evaluating the performance of other models, and the results showed that the predicted value of the optimal ARIMA (0, 1, 1) (0, 1, 1)_12_ model was essentially consistent with the actual value, but there was still a certain gap. The possible reason is that the real-time series are generally a combination of linear and nonlinear relationships. The ARIMA model can extract the linear components of the time series, but it loses the nonlinear information in the residual. An artificial neural network is an emerging technology that can imitate the learning and reasoning process of the human brain and nervous system and has a nonlinear mapping ability. In this paper, we used human brucellosis data to compare the performance of the ARIMA-BPNN, ARIMA-ERNN and ARIMA models in fitting and prediction. The study found that compared with the ARIMA model, the MAE, MSE and MAPE of the ARIMA-ERNN and ARIMA-BPNN models had different degrees of decline in terms of fitting and prediction performance. The fitting and prediction performances of the combined model were better than those of the single ARIMA model, consistent with the research results of other scholars [46]. The combined model compensates for the lack of nonlinear mapping ability of the ARIMA model and modifies the predicted value of the ARIMA model. Compared with the ARIMA-BPNN, the MAE, MSE and MAPE of the ARIMA-ERNN model also decreased to different degrees. The ARIMA-ERNN model had the best effect in predicting the incidence of human brucellosis and was superior to the other two models. The reason may be that the BPNN regards the prediction process as static system modelling, while ERNN is based on the structure of the BPNN and uses the receiving layer to provide its function of mapping dynamic characteristics. Therefore, ERNN can better adapt to event changes and fit time series, thus achieving the highest prediction accuracy.

To the best of our knowledge, this is the only study to explore a combined model of ARIMA and ERNN for predicting the incidence of human brucellosis. Its advantage is that the ARIMA-ERNN model combines the advantages of ARIMA in linear features and a neuron network in nonlinear features and enhances the capability of a single ARIMA while retaining the advantage of its simplicity in utilizing only incidence time series data as input. Second, based on the structure of the BPNN, the ERNN adds a corresponding receiving layer in the hidden layer to provide its dynamic memory and strong sensitivity to time series, which are more suitable for analysing human brucellosis. Third, the use of the ARIMA-ERNN model contributes to rational allocation of limited public health resources and the early prevention and control of human brucellosis.

Nevertheless, there are also some limitations. First, the epidemic pattern and incidence of human brucellosis are different in different areas. Whether the ARIMA-ERNN model is suitable for other regions needs further study [47]. Second, the incidence of human brucellosis is vulnerable to many factors [19]. This study used only monthly incidence data, which may have impacted the performance of the models. Third, only two combinatorial models are established in this study, and the superiority of the ARIMA-ERNN model and other models remains to be verified. In the future, we will incorporate the influencing factors of human brucellosis into the prediction model and compare the ARIMA-ERNN with other models.

## Conclusions

In this study, the time series of human brucellosis in Shanxi Province from 2007 to 2017 showed obvious seasonal characteristics and a trend of first increasing and then decreasing. The fitting and prediction performances of the ARIMA-ERNN model were better than those of the ARIMA-BPNN and ARIMA models, and the ARIMA-BPNN model was better than the ARIMA model. The ARIMA-ERNN model was more suitable for predicting the incidence of human brucellosis than the ARIMA and ARIMA-BPNN models.

## Data Availability

The datasets analysed during the current study are not publicly available because they are infectious disease data but are available from the corresponding author upon reasonable request.

## Abbreviations

STL: Seasonal-trend decomposition using Loess
ARIMA model: Autoregressive integrated moving average model
ARIMA-BPNN: A combined model of ARIMA and back propagation neural network
ARIMA-ERNN: A combined model of ARIMA and Elman recurrent neural network
MAE: Mean absolute error
MSE: Mean squared error
MAPE: Mean absolute percentage error
BPNN: Back Propagation Neural Network
MARS: Multivariate Adaptive Regression Splines
RF: Random Forest
MLP: Multilayer Perceptron networks
SVM: Support Vector Machines
RBF: Radial Basis Function
ERNN: Elman recurrent neural network
ACF: Autocorrelation function
PACF: Partial autocorrelation function
ADF: Augmented Dickey-Fuller
AIC: Akaike information criterion
SBC: Schwarz Bayesian information criterion
R^2^: The coefficient of decision
MLE: Maximum likelihood estimation
ANNs: Artificial neural networks
